# Comparative Analysis of piRNA Profiles Helps to Elucidate Cryoinjury Between Giant Panda and Boar Sperm During Cryopreservation

**DOI:** 10.3389/fvets.2021.635013

**Published:** 2021-04-22

**Authors:** Yihan Wang, Yingmin Zhou, Malik Ahsan Ali, Jiaman Zhang, Wencan Wang, Yan Huang, Bo Luo, Heming Zhang, Ziyue Qin, Yan Zhang, Ming Zhang, Guangbin Zhou, Changjun Zeng

**Affiliations:** ^1^College of Animal Sciences and Technology and Farm Animal Genetic Resources Exploration and Innovation Key Laboratory of Sichuan Province, Sichuan Agricultural University, Chengdu, China; ^2^China Conservation and Research Center for the Giant Panda, Chengdu, China; ^3^Department of Theriogenology, Riphah College of Veterinary Sciences, Lahore, Pakistan

**Keywords:** piRNAs, sperm cryopreservation, freezability, boar, giant panda

## Abstract

Cryopreservation induces sperm cryoinjuries, including physiological and functional changes. However, the molecular mechanisms of sperm cryoinjury and cryoresistance are still unknown. Cryoresistance or the freeze tolerance of sperm varies across species, and boar sperm is more susceptible to cold stress. Contrary to boar sperm, giant panda sperm appears to be strongly freeze-tolerant and is capable of surviving repeated cycles of freeze-thawing. In this study, differentially expressed (DE) PIWI-interacting RNAs (piRNAs) of fresh and frozen-thawed sperm with different freeze tolerance capacity from giant panda and boar were evaluated. The results showed that 1,160 (22 downregulated and 1,138 upregulated) and 384 (110 upregulated and 274 downregulated) DE piRNAs were identified in giant panda and boar sperm, respectively. Gene ontology (GO) enrichment analysis revealed that the target DE messenger RNAs (mRNAs) of DE piRNAs were mainly enriched in biological regulation, cellular, and metabolic processes in giant panda and boar sperm. Moreover, Kyoto Encyclopedia of Genes and Genomes (KEGG) analysis indicated that the target DE mRNAs of DE piRNAs were only distributed in DNA replication and the cyclic adenosine monophosphate (cAMP) signaling pathway in giant panda, but the cAMP, cyclic guanosine monophosphate (cGMP), and mitogen-activated protein kinase (MAPK) signaling pathways in boar sperm were considered as part of the olfactory transduction pathway. In conclusion, we speculated that the difference in the piRNA profiles and the DE piRNAs involved in the cAMP signaling pathway in boar and giant panda may have contributed to the different freeze tolerance capacities between giant panda and boar sperm, which helps to elucidate the molecular mechanism behind sperm cryoinjury and cryoresistance.

## Introduction

Sperm cryopreservation is widely used to manage and preserve male fertility in human and domestic animals (1). Then, artificial insemination (AI) is extensively employed with frozen-thawed sperm to enhance the rate of genetic improvement, especially in cattle (2). However, sperm cryoresistance or freeze tolerance and the post-thawed sperm quality vary across species. Less than 1% AI with frozen-thawed boar sperm was carried out due to the low conception rate and litter size (3, 4). It is well-known that various factors during cryopreservation, including rapid temperature transitions, osmotic stress, and ice crystal formation, affect the post-thaw quality of semen (5). Furthermore, the transcriptomics, epigenetics, and proteomics of sperm were also modified during cryopreservation (6–8). Despite the extensive progress that has been achieved in optimizing the cryopreservation process through the selection of friendly cryoprotectants and the design of better freezing and thawing procedures to ameliorate cryodamage, the underlying mechanisms of freeze tolerance or freezability involved in cryopreservation have not been completely elucidated yet.

Compared with other mammals' sperm, the higher level of phospholipids and the lower level of cholesterol in the plasma membrane of boar sperm contribute to the susceptibility to cold shock or cold stress (9). Cold shock causes the rearrangement of phospholipids, destruction of acrosomal integrity, and functional damage to ion transporters and channels in sperm (9, 10). However, compared with boar sperm, giant panda sperm shows a higher freeze tolerance capacity and can sustain repeated freeze–thaw cycles (11). Cryopreservation has no significant impact on sperm viability and motility, and the acrosome integrity and functional capacitation of giant panda sperm were also not affected after repeated freeze–thaw cycles (12). Our previous studies have shown that the transcriptomic profiles were significantly different between boar and giant panda sperm during cryopreservation (13). Furthermore, comparative analysis of the transcriptomic modifications between boar and giant panda sperm during cryopreservation indicated that differentially expressed (DE) messenger RNAs (mRNAs) were mainly distributed in inflammatory-related pathways, the cytokine–cytokine receptor interaction pathway, and membrane signal transduction-related pathways (14). These previous studies demonstrated that cryopreservation induces different transcriptomic modifications and may explain why sperm with different freeze tolerance or cryoresistance capacities are susceptible to cold stress.

PIWI-interacting RNAs (piRNAs) are small non-coding RNAs which are germline-specific and are required to protect genomic integrity from deleterious effects and to preserve RNA homeostasis during male gametogenesis; they are also associated with sperm morphology, motility, and fertility (15). The expression of piRNAs in human sperm was correlated with the sperm concentration and fertilization rate (16). Moreover, a panel of piRNAs discovered in seminal plasma can serve as fertility or infertility markers in males (17). Recently, 79 putative piRNAs were found to be differentially expressed between low and high motile bovine sperm after cryopreservation (18). Therefore, we speculated that piRNAs may be involved in post-thawed sperm cryoinjury or cryoresistance, motility, and fertility during cryopreservation. Thus, in this study, we first evaluated the differences in the piRNA profiles of fresh and frozen-thawed boar and giant panda sperm, which will help to uncover the underlying molecular mechanisms of sperm cryoresistance and freeze tolerance and improve post-thawed sperm quality and fertility.

## Materials and Methods

### Ethical Statement, Semen Collection, and Treatment

Fresh ejaculates from five sexually mature giant pandas with normal physiological parameters were obtained by electrical stimulation from the Bifengxia Base of China Conservation and Research Center for the Giant Panda (Yaán, Sichuan, China) according to a previous protocol (11). Briefly, giant pandas were anesthetized by an intramuscular injection of 10 mg/kg ketamine HCl and maintained with 0–5% isofluorane gas. Electroejaculation was conducted by using an electroejaculator (Boring, OR, USA); the period of electrical stimuli (2–8 V, repeated three times) was 2 s following an intermittent break of 2 s. When penile erection occurs during stimulation, semen was collected into a sterile glass container. Fresh ejaculates from 11 boars were collected with the glove-handed technique. All procedures were carried out while strictly following the Regulations of the Administration of Affairs Concerning Experimental Animals (Ministry of Science and Technology, China, revised in June 2004) and were accredited by the Institutional Animal Care and Use Committee in the College of Animal Science and Technology, Sichuan Agricultural University, Sichuan, China (under permit no. 2019202012).

All ejaculates from giant panda and boar were pooled separately and two equal groups were generated (fresh sperm and cryopreserved sperm). Direct RNA extraction was performed with fresh sperms, and the other group was cryopreserved according to a previously procedure (19). Briefly, TES–Tris (TEST) egg yolk buffer was used to dilute the giant panda sperm (Irvine Scientific, Santa Ana, CA) to obtain 5% concentration of glycerol. This diluted material was filled into 0.25-ml semen straws and gradually cooled to 4°C in 4 h, then kept at 7.5 cm for 1 min over liquid nitrogen (LN) to obtain the cooling rate of −40°C/min and then at 2.5 cm for 1 min above LN (approximate cooling rate was −100°C/min), before plunging in LN until further processing. Thawing was performed by immersing the semen straws for 30 s in a water bath with constant temperature of 37°C. Semen was diluted with an equal volume of Ham's F10 (HF10) containing 5% fetal calf serum and 25 mM HEPES. Boar sperm was cryopreserved according to the following procedure; firstly, the sperm was centrifuged (for 5 min at 1,800 rpm and 17°C) and then diluted with a lactose–egg yolk (LEY) extender containing 10 ml hen's egg yolk and 40 ml 11% β-lactose. Secondly, the sperm and the extender mixture were cooled to 4°C (at 0.2°C/min), and further dilution with LEY was performed to obtain a final 3% concentration of glycerol. Lastly, the 0.25-ml semen straws (FHK, Tokyo, Japan) were loaded with this mixture, sealed, and kept 3 cm above LN for 10 min before being submerged into it until future use.

### RNA Extraction, Library Preparation, and Sequencing

Before RNA extraction, seminal plasma was removed from all the samples by washing with RNase-free water three times. Then, 0.5% Triton (X-100) was employed in accordance with a previous study (16) to minimize the somatic cell count as they hinder the spermatic RNA extraction process. Then, the TRIzol LS Reagent kit (Invitrogen, Carlsbad, CA, USA) was utilized to extract total RNA from all sperm samples according to the manufacturer's instructions. The RNA samples were pooled together equally in their respective groups before constructing RNA libraries. Furthermore, a Nanodrop (Thermo Fisher Scientific, Wilmington, DE, USA) equipment was used to determine the purity and concentration of the RNA and an Agilent 2100 Bioanalyzer (Agilent Technologies, Santa Clara, CA, USA) was employed to check its integrity. Then, a NEBNext Poly(A) mRNA Magnetic Isolation Module (NEB, E7490, Ipswich, MA, USA) was utilized to isolate mRNA. The small RNA libraries were built by using the NEB Next Ultra RNA Library Prep Kit for Illumina (NEB, E7530, Ipswich, MA, USA) and the NEBNext Multiplex Oligos for Illumina (NEB, E7500, Ipswich, MA, USA) according to the manufacturer's guidelines. After confirming the quality using Qubit 2.0 and the Agilent Bioanalyzer 2100 system (Agilent Technologies), all libraries were sequenced with the Illumina Hiseq 2500 platform (Illumina, San Diego, CA, USA).

### piRNA Identification and Expression Analysis

After removal of low-quality, poly-N, and adapter-containing reads and sequences with <18 or >34 nt, clean reads were acquired. The sequence alignments of giant panda and pig were carried out with their reference genomes (ftp://ftp.ncbi.nlm.nih.gov/genomes/all/GCF/000/004/335/GCF_000004335.2_AilMel_1.0 and ftp://ftp.ensembl.org/pub/release-75/fasta/sus_scrofa/, respectively). Furthermore, to compare the clean reads with the Silva database, Rfam database, Repbase, and the GtRNAdb database and filtering out non-coding RNAs (ncRNAs) such as ribosomal RNA (rRNA), transport RNA (tRNA), small nuclear RNA (snRNA), small nucleolar RNA (snoRNA), and repetitive sequences, Bowtie analysis was performed (20). Novel and known piRNAs were sorted out by comparing the obtained piRNA sequences with miRbase RNA sequences using proTRAC (21). Differential expression of piRNAs in the fresh and frozen-thawed groups was determined with the DESeq R package (v. 1.10.1) based on the reads per kilobase million (TPM) and fragments per kilobase million (FPKM) algorithms (22). The piRNAs between both sperm groups were analyzed by iDEG (23), and those with adjusted *p* < 0.01 and absolute value of log2 fold change (FC) >1 were classified as DE piRNAs. Then, hierarchical clustering analysis was performed by R heatmap.2 on the selected DE piRNAs; piRNAs with similar expressions were clustered based on the log10(TPM + 1) value.

### piRNA Target Prediction, GO, and KEGG Enrichment Analyses

The prediction of potential piRNA targets was performed by BLAST with non-redundant (NR) (20), Gene Ontology (GO) (20), Kyoto Encyclopedia of Genes and Genomes (KEGG) (24), and EuKaryotic Orthologous Group (KOG) (25) databases to obtain annotation information of the target genes. KEGG pathways and GO enriched in predicted DE piRNA target genes were elucidated using KOBAS software (26) and the GOseqR package (27), respectively.

### Comparison of DE piRNAs in Fresh and Post-thawed Boar and Giant Panda Sperm During Cryopreservation

piRBase (http://www.regulatoryrna.org/database/piRNA/) was used to browse the common piRNAs and annotations. The homology of piRNAs was predicted between various species by considering the similarity and conserved sequences of the piRNAs to determine the piRNAs. The software Python 2.7 was used for comparing the sequence similarities of the DE piRNAs in giant panda sperm and boar sperm. During sequence alignment, 1–18 bases were perfectly matched; one mismatch base was allowed after the 19th base to select the best pairing sequence (28).

### Statistical Analysis

All data were shown as the means ± SEM. SPSS (v. 20.0) with independent samples *t* test was used to determine statistical differences, and *p* < 0.05 were considered as statistically significant.

## Results

### piRNA Profiles of Fresh and Cryopreserved Boar and Giant Panda Sperm

A total of 16,980,071 and 19,571,331 raw reads were obtained from fresh and cryopreserved sperm groups of giant pandas, respectively. Similarly, respective boar sperm groups generated 18,956,444 and 16,507,275 raw reads. After removal of low-quality reads, ploy-N, adapter, and sequences with <24 or >32 nt, 519,311 and 4,488,163 clean reads were generated in respective fresh and frozen-thawed giant panda sperm. Similarly, 9,031,512 and 7,188,244 clean reads were generated in fresh and frozen-thawed boar sperm, respectively (Table 1). The 24-nt (21.76%) and 31-nt (34.21%) piRNAs were the most abundant in fresh and frozen-thawed giant panda sperms, respectively. Similarly, the 30-nt (25%) and 32-nt (1.96%) piRNAs showed the highest and the lowest respective abundances, respectively, in boar sperm.

**Table 1:** Overview of piRNA sequencing of fresh and frozen-thawed sperm in giant panda and boar.

**Species**	**Group**	**Raw reads**	**Clean reads**	**Mapped reads**	**Mapped ratio (%)**
Giant panda	Fresh sperm	16,980,071	519,311	85,586	16.48
	Post-thawed sperm	19,571,331	4,488,163	325,288	7.2
Boar	Fresh sperm	18,956,444	9,031,512	2,988,336	15.76
	Post-thawed sperm	16,507,275	7,188,244	2,087,711	13.0

**Figure 1:**
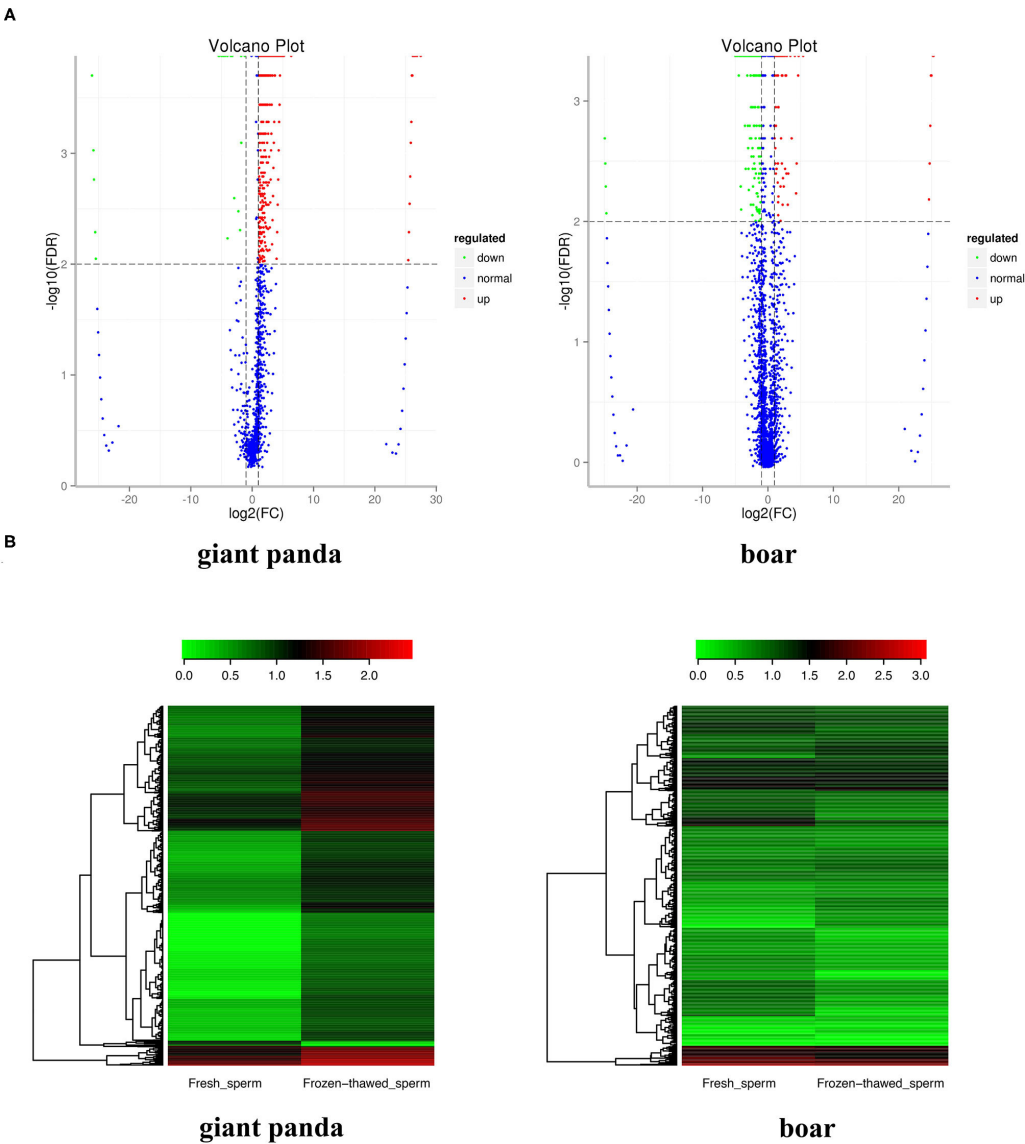
Volcano plot and clustering analysis of differentially expressed PIWI-interacting RNAs (DE piRNAs) in fresh and frozen-thawed giant panda and boar sperm. **(A)** Volcano plot of DE piRNAs in fresh and frozen-thawed giant panda sperm. *Blue dots* represent normal expressed, *green dots* represent the downregulated, and *red dots* represent the upregulated piRNAs. **(B)** Heat maps of the cluster analysis of piRNAs. *Red* indicates high expression while *green* means low expression of piRNAs.

A total of 88 (containing 116,706 piRNAs) and 133 (containing 21,5835 piRNAs) piRNA clusters were identified after mapping to the designated reference genomes of giant panda and boar sperm, respectively. Compared to the 125,435 and 112,708 piRNAs expressed in fresh and frozen-thawed boar sperm, respectively, 49,393 and 87,670 piRNAs were expressed in fresh and frozen-thawed giant panda sperm, respectively. Differential analysis depicted the differential expression of 1,160 piRNAs (1,138 upregulated and 22 downregulated) between fresh and frozen-thawed giant panda sperm (Figure 1A). In contrast to the giant panda sperm, 384 DE piRNAs (110 upregulated and 274 downregulated) were identified in boar sperm (Figure 1A). Moreover, hierarchical clustering analysis was performed for the clustering of all DE piRNAs (Figure 1B).

### Combined Analysis of piRNAs and Target mRNAs in Boar and Giant Panda Sperm

**Figure 2:**
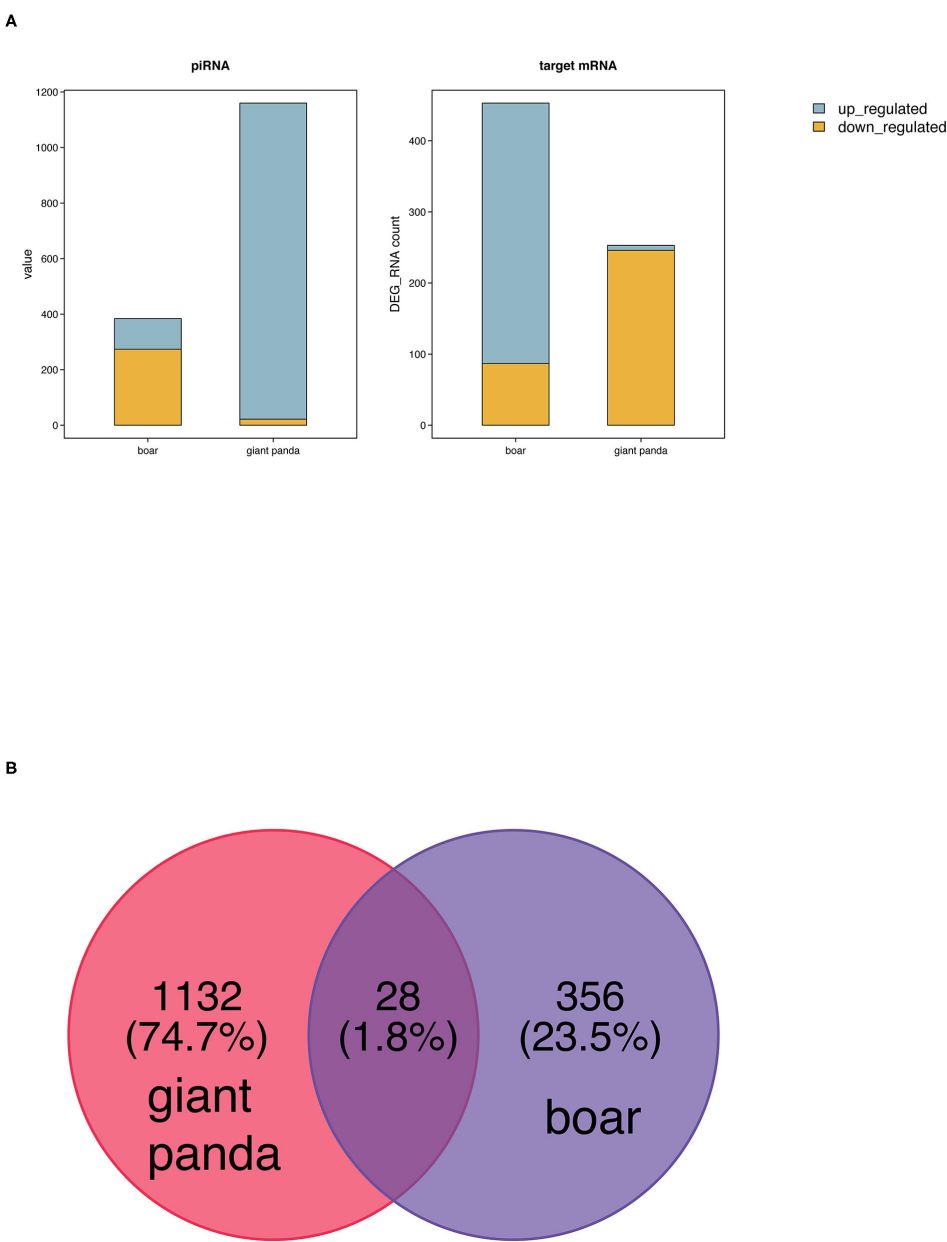
Comparative analysis of differentially expressed PIWI-interacting RNAs (DE piRNAs) in fresh and frozen-thawed giant panda and boar sperm. **(A)** Comparison of DE piRNAs and target DE mRNAs. **(B)** Unique and common DE piRNAs.

Two hundred fifty-three (seven upregulated and 246 downregulated) and 453 target DE mRNAs (366 upregulated and 87 downregulated) of the DE piRNAs were obtained between fresh and post-thawed sperm in giant panda and boar, respectively (Figure 2A). Twenty-eight DE piRNAs were identified to be the common piRNAs by joint analysis of the DE piRNAs of giant panda and boar sperm (Figure 2B). Therefore, 1,132 and 356 DE piRNAs were selected and regarded as the unique piRNAs in giant panda and boar sperm, respectively (Data Sheet 1, 2). Based on the similarity and conservation of the piRNA sequences, 28 DE piRNAs were considered as the homologous piRNAs between giant panda and boar sperm according to the piRBase database (Data Sheet 3). However, no target DE mRNAs were found for these common DE piRNAs.

### Comparative GO and KEGG Analysis of DE piRNAs in Giant Panda and Boar Sperm

**Figure 3:**
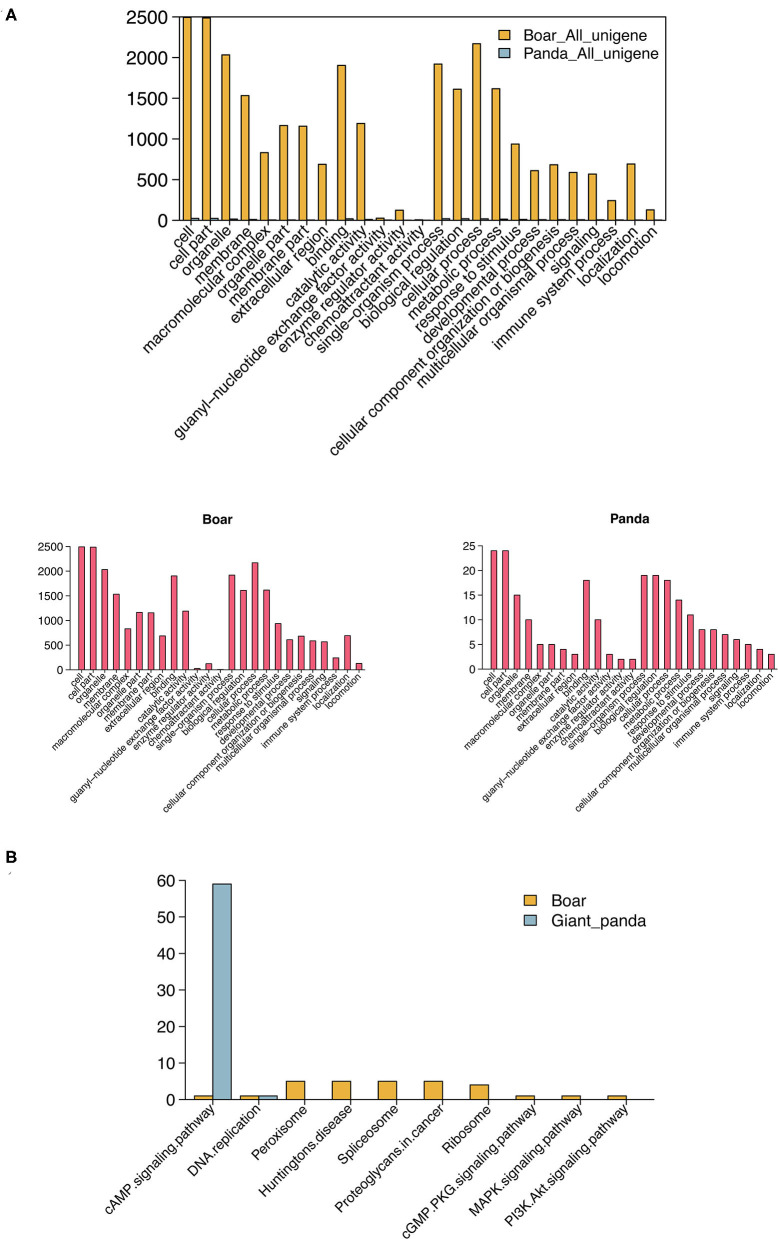
Gene Ontology (GO) and Kyoto Encyclopedia of Genes and Genomes (KEGG) analysis of differentially expressed PIWI-interacting RNAs (DE piRNAs) in giant panda and boar sperm. **(A)** GO analysis of the target DE messenger RNAs (mRNAs) of DE piRNAs. **(B)** Top 10 KEGG pathways of the target DE mRNAs of DE piRNAs.

GO enrichment analysis showed that 106 and 3,251 target DE mRNAs of the DE piRNAs were annotated with 41 and 59 GO terms in giant panda and boar sperm, respectively. Most of the target mRNAs of the DE piRNAs were seen to be distributed in cell, cell part, binding and biological regulation, and metabolic terms in giant panda and boar sperm, which are strictly associated with the structural and functional modifications of sperm. The GO term distributions of the target DE mRNAs of DE piRNAs were significantly different in fresh and frozen-thawed giant panda and boar sperm (Figure 3A).

Notably, most of the target DE mRNAs of DE piRNAs were distributed in the cyclic adenosine monophosphate (cAMP) signaling pathway in giant panda sperm, except for DNA replication (Figure 3B). However, the target mRNAs of the DE piRNAs in boar sperm were mainly distributed in the peroxisome and spliceosome, followed by the membrane-related pathway, such as the cAMP, cyclic guanosine monophosphate (cGMP), mitogen-activated protein kinase (MAPK), and PI3K–Akt signaling pathways. Moreover, the cAMP pathway was found in both giant panda and boar sperm, but was extremely enriched in giant panda sperm. Further analysis indicated that DE piRNAs involved in the cAMP signaling pathway may regulate the post-thawed sperm function by targeting cyclic nucleotide-gated (CNG) ion channel-related genes.

## Discussion

It is well-known that differences in the size, shape, and the lipid–protein composition of sperm across various species result in different sensitivities to freezing (29, 30). Esmaeili and colleagues have demonstrated that cryotolerance shows a relation to the ratio of polyunsaturated fatty acids (PUFAs) (omega-3/omega-6) (30). The plasma membrane of boar sperm contains a higher concentration of phospholipids and a lower concentration of cholesterol (9). In addition, the head size of boar sperm is larger than that of the giant panda and is more sensitive to freezing (31). Sperm with smaller heads are usually less cryopreservation-sensitive; thus, the freeze tolerance capacity of giant panda sperm is higher than that of boar sperm after cryopreservation (14).

Nowadays, electroejaculation is the preferred method to collect semen from giant panda. Previous studies have reported that electroejaculation may have an impact on semen parameters, increasing semen osmolarity, disrupting plasma membrane integrity, acrosomal damage, and acrosomal exocytosis (32, 33). However, the sperm morphology remained within acceptable standards (34). Compared with the quality parameters of fresh feline ejaculates collected using three different techniques—urethral catheterization after medetomidine administration, electroejaculation, and epididymal slicing after orchiectomy—the highest quality semen parameters were achieved using electroejaculation (35). Spindler et al. reported that most sperm of giant panda were morphologically normal using electroejaculation, and the sperm parameters (seminal volume, concentration, initial motility, acrosomal integrity, etc.) were consistent with previous reports (11). Therefore, the fertility of frozen-thawed giant panda sperm will be similar to that following the use of fresh sperm (11).

The process of freeze–thawing induces apoptotic-like changes in sperm, and these changes may affect the plasma membrane and acrosomal activity (36) and the mitochondrial activity (37) and also cause abnormal expressions of genes and proteins associated with cryoinjury (38). Moreover, sperm genomic epigenetic elements may be altered during cryopreservation. Previous studies have demonstrated that some genes play critical roles in freezing, such as PRM1, FSHB, ADD1, ARNT, and SNORD116/PWSAS (39, 40). Some proteins, such as TPI1, ACRBP, HSP90AA1, and PHGPx, were proven to be markers of sperm cryoresistance (38, 41, 42). Furthermore, certain mRNA transcripts encoding related proteins were affected during cryopreservation; for instance, PRM1 mRNA transcripts were reduced in boar, cattle, and human sperm (43–45). Beyond that, some sperm mRNA transcripts associated with early embryo development were downregulated in embryos fertilized with frozen horse sperm compared to those with fresh sperm (46). In fact, some microRNAs (miRNAs) associated with cryopreservation, or named CryomiRs, may affect the expressions of the mRNA transcripts during cryopreservation, which ultimately affects the expressions of genes and proteins associated with sperm metabolism and apoptosis (14, 47).

According to our previous study, the DE miRNAs and target mRNAs of giant panda sperm were mainly enriched in olfactory transduction pathways, including the cAMP and cGMP signaling pathways (14). In the present study, we found that the target DE mRNAs of DE piRNAs in giant panda sperm were mainly distributed in the cAMP signaling pathway and partially involved in DNA replication. Similarly, few targets of the DE piRNAs in boar sperm were also enriched in the cAMP signaling pathway, but the ratio was much lower than that of giant panda sperm. Therefore, we speculated that the 1,132 specific piRNAs involved in the cAMP signaling pathway in giant panda sperm may be closely related to the freeze tolerance of sperm. Therefore, we speculated that cryopreservation can affect the expression levels of olfactory transduction pathway-related genes and is probably involved in the regulation of capacitation, motility, fertility, and even the freeze tolerance of post-thawed sperm. However, the regulatory mechanism of the olfactory transduction signaling pathway on post-thawed sperm is still unknown.

It is well-known that olfactory receptors or odorant receptors are associated with sperm motility and chemotaxis. In the olfactory transduction pathway, after the attachment of odorant molecules with the G protein-coupled receptor (GPCR) in sperm, the concentration of cAMP increases, leading to the opening of CNG ion channels (48). Notably, CNG channels play an important role in the regulation of the intracellular Ca^2+^ level, which causes influx of Ca^2+^, and then induce sperm hyperactivity (49). In mature sperm, cAMP binding with a target protein is essential for those events during sperm capacitation, including sperm plasma membrane hyperactivation (50, 51), tyrosine phosphorylation (52), and increasing intracellular Ca^2+^ and pH (53–55). It was demonstrated that the intracellular concentrations of cAMP and Ca^2+^ play a primary role in sperm capacitation, motility, acrosomal reaction, lipid remodeling, and hyperpolarization of the plasma membrane (55–58). Furthermore, cAMP is known to be an important second messenger for steroid (hormones) biosynthesis, and the specific role of its downstream protein kinase A (PKA) pathway is regulating steroid biosynthesis (59). Steroid hormones induce sperm capacitation and acrosomal response (60). cAMP–PKA signaling pathways induce steroid biosynthesis in stromal cells by activating certain transcription factors, such as CREB, CREM, and GATA4, and regulating the expressions of downstream target proteins (58, 61). In addition, the synthesis of cAMP also activates a Ca^2+^ signal regulated by PKA or protein kinase C (PKC), which upregulates Nur77 expression, and causes StAR transcription, promoting steroid hormone biosynthesis (62). Previous studies indicated that the intracellular Ca^2+^, 1,2-diacylglycerol (DAG), and cAMP levels in buffalo sperm were increased significantly after cryopreservation as compared to fresh ejaculates, and the addition of taurine or trehalose reduced the extent of capacitation-like changes in buffalo sperm (56). Likewise, cryopreservation negatively affected the PKA and AMP-activated protein kinase (AMPK) activity in Atlantic salmon sperm (63), and when AMPK was inhibited, the sperm motility decreased accordingly. In this study, the target mRNAs of the DE piRNAs in giant panda sperm are mainly enriched in the cAMP pathway, which indicates that cAMP and calcium may be associated with frozen-thawed sperm quality of giant panda. Differences in the cAMP pathway-related piRNAs and mRNAs between the giant panda and boar sperm may have contributed to sperm cryotolerance. Therefore, our study first revealed that piRNAs might be regulating the cAMP signaling pathway to regulate post-thawed sperm quality, which provides new insights into the cryoinjury, cryoresistance, or the freeze tolerance mechanisms of sperm varying across species. Future exploration should focus on the biological roles of these DE piRNAs in sperm freeze tolerance or cryoresistance and their association with post-thawed sperm quality, which may provide some insights regarding the molecular mechanisms of cryoinjury.

## Conclusion

In this study, we first conducted a comparative analysis of the piRNAs and target mRNAs between giant panda sperm and boar sperm during cryopreservation. The differentially expressed piRNAs and their target DE mRNAs are mainly involved in the cAMP signaling pathway and DNA replication, which indicated that these piRNAs play a critical role in sperm cryoresistance and cryoinjury during cryopreservation. Our study provides new insights into the cryoinjury, cryoresistance, or freeze tolerance mechanisms of sperm varying across species.

## Data Availability Statement

The datasets presented in this study can be found in online repositories. The names of the repository/repositories and accession number(s) can be found below: Gene Expression Omnibus, GSE163128.

## Ethics Statement

The animal study was reviewed and approved by the Regulations of the Administration of Affairs Concerning Experimental Animals (Ministry of Science and Technology, China, revised in June 2004) the Institutional Animal Care and Use Committee in the College of Animal Science and Technology, Sichuan Agricultural University, Sichuan, China, under permit No: 2019202012. Written informed consent was obtained from the owners for the participation of their animals in this study.

## Author Contributions

YW, YZho, MA, JZ, WW, and ZQ collected samples, performed the experiments, analyzed the data, and drafted the manuscript. BL, YH, and HZ contributed to samples collection, data analysis, and revised the manuscript. MZ, GZ, and YZha revised the manuscript critically and given final approval to be published. CZ granted, concept, designed the experiment and revised, given final approval version of the manuscript to be published. All authors reviewed and approved the final manuscript.

## Conflict of Interest

The authors declare that the research was conducted in the absence of any commercial or financial relationships that could be construed as a potential conflict of interest.

## Supplementary Material

Data Sheet 1 All DE piRNAs in giant panda and boar sperm.

Data Sheet 2 DE piRNAs and their target DE mRNAs in giant panda and boar sperm.

Data Sheet 3 The common DE piRNAs in giant p**a**nda and boar sperm.
